# Liquid Polycaprolactone (PCL) for Reversing PXE's Skin Laxity of Inner Thighs and Knees: A Case Report

**DOI:** 10.1111/jocd.16713

**Published:** 2024-12-20

**Authors:** Emmanouil Dimonitsas, Agni Albanopoulou, Foteini Neamonitou, Eleftheria Lakiotaki, Penelope Korkolopoulou, Maria Gazouli

**Affiliations:** ^1^ Dermatology and Plastic Surgery Clinic “Skin Medical Secrets” Athens Greece; ^2^ Department of Plastic and Reconstructive Surgery “Saint‐Savvas” Hospital, Anti‐Cancer Institute Athens Greece; ^3^ Department of Pathology National and Kapodistrian University of Athens Athens Greece; ^4^ Department of Biology and Nanomedicine National and Kapodistrian University of Athens Athens Greece

**Keywords:** Collagen synthesis, epidermal stimulation, Fibroblasts, Rejuvenation

## Abstract

**Background/Aim:**

Pseudoxanthoma elasticum (PXE) is a genetic connective tissue disorder that affects the skin with limited treatment options. A recent technology employing particle‐free polycaprolactone (PCL) has shown promising results in treating inner thighs and kness of a 27‐year‐old female patient. This article provides a case report along with our detailed treatment protocol based on the efficacy of PCL in reversing skin laxity that can be easily incorporated into the therapeutic approaches for patients with PXE.

**Methods:**

The pinch test for clinical assessment, photographic documentation as well as satisfaction questionnaire were used in order to evaluate the improvement observed in skin laxity.

**Results:**

After the finish of the protocol, the Pinch test score was diminished from 4 to 2, and the depth of the wrinkles was obviously improved in the photos. All the above, together with the high score of the patient satisfaction questionnaire, verified the efficacy of this novel PCL protocol in reversing PXE‘s skin laxity.

**Conclusion:**

Fully liquid PCL appears to outperform other bio‐stimulators in improving skin quality in PXE, offering longer‐lasting results and a high safety profile. However, further clinical trials with long‐term follow‐up are required to confirm its efficacy in PXE‘s skin laxity.

## 1 Background

Pseudoxanthoma elasticum (PXE) is an inherited genetic (autosomal dominant or recessive) connective tissue disorder characterized by abnormal calcification and fragmentation of the elastic tissue network, mainly of the skin [1]. Manifestations are also apparent from other systems such as the gastrointestinal, cardiovascular, and ocular [2]. The diagnosis is most often in the second decade of life with a gradual progression in the next years [3]. Its cutaneous findings include yellowish papules, relatively small (1–3 mm), accompanied by skin laxity, especially on flexural areas (antecubital, popliteal, inguinal, cervical. axillary folds, and periumbilical). The area of the inner thighs often exhibits dermal changes characterized by increasing redundancy, diminished tautness, heightened rigidity, and, ultimately, the development of wrinkles [4].

Specific criteria have been established for diagnosing PXE [5] based on molecular (two pathogenic mutations in the ABCC6 gene), clinical (ocular and skin findings), and histopathological characteristics [5]. Despite the standardized diagnosis over the years, treatment options for the skin manifestation of PXE are limited, and the results are often unsatisfactory for PXE patients. There are no standardized treatment protocols for the PXE's skin manifestations, and all the suggested strategies for the improvement of skin quality are in the context of case studies. Numerous cases have been treated, and the outcomes of these treatments have been documented in international journals.

The treatments include ablative and non‐ablative lasers, topical retinoid therapies, micro‐needling devices, and bovine collagen injectables [6, 7, 8]. However, the results in most cases were temporary, leading many patients to seek surgical treatments [9]. A comprehensive three‐step Greek protocol designed to address neck skin tightening involved a combination of laser skin resurfacing with equine collagen booster type I and platelet‐rich plasma (PRP). While this approach improved skin texture, it was noted that the firming effects were transient, lasting for 6 months. Subsequently, the skin quality regressed to its prior state over time [10].

The primary objective is to counteract the looseness associated with this rare disease through dermal bio‐reconstruction. A novel biostimulator is utilized to encourage collagen synthesis by fibroblasts. Polycaprolactone (PCL), a particle‐free, fully liquid polymer that readily dissolves in water, shows promise in efficiently stimulating collagen production from fibroblasts in the deep dermis [11].

The biocompatibility and behavior of PCL have been well‐documented since the 1980s. The initial PCL fillers on the market consisted of noncross‐linked PCL microspheres, sized between 20 and 50 μm, suspended in an aqueous carboxymethylcellulose (CMC) gel. However, these PCL particles were not evenly distributed in the tissue, leading to mild swelling and redness immediately after treatment, as well as granuloma formation in many cases, occurring 6 months later [12].

To address these limitations, DEXLEVO Inc. has developed a new dermal bio stimulator made of particle‐free PCL (GOURI). This patented technology, known as Collagenesis‐Enabled Solubilized Active and Biodegradable Polymer Technology (CESABP), ensures excellent spreadability of the product due to its fully liquefied PCL composition. Furthermore, the absence of micro‐particles in the product ensures safety by eliminating the possibility of vascular occlusion and long‐term granulomatous reactions [13].

Fully liquid PCL with no severe or long‐term side effects and complications registered until now creates a strong stimulation of impaired fibroblasts to produce new collagen and elastin fibers. With the absence of micro particles, PCL itself creates an appropriate microenvironment in the dermis for fibroblasts with the necessary signals to repair skin imperfections of PXE from the inside in order to have obvious skin quality improvement from the outside.

## 2 Objective

In this study, we aimed to propose liquid polycaprolactone (PCL) as a new treatment for improving the skin condition of a young woman with PXE.

## 3 Materials and Methods

The dermal filler, developed by Dexlevo Inc. in Seoul, Korea, is a particle‐free PCL solution dissolved in water. The procedure involved using eight 1 mL syringes of the PCL‐based dermal filler (GOURI), six syringes administered to the inner thighs and two syringes to the knees during each session.

The dosage used for this protocol was not recommended officially by the manufacturer. Therefore, it was calculated based on the standardized protocols for the face and neck. For each of the mentioned protocols, a total of two syringes are recommended per session. As the inner thighs' area to be treated in this case was almost three times larger than the face's area, we calculated that a total of six syringes would be sufficient to obtain the expected efficacy. Using the same approach, two syringes (one per each side) were used for the knees, as the total surface that we wanted to cover was almost the same as the face's.

For the inner thighs, three entry points were used on each side, drawn on a horizontal line beginning 3 cm below the pubic bone and ending up at the adductor tubercle, with 3 cm intervals between them, and one syringe was injected at each point. As for the knees, the injection site was 2 cm above the knee joint on the inner side.

To address the thighs, the procedure commenced with the administration of local anesthesia (2% lidocaine) into the designated entry points. Subsequently, a 25‐gauge, 50 mm cannula was utilized to meticulously introduce the substance into the subcutaneous tissue proximal to the dermis. Ultimately, a retrograde technique with several injection vectors was employed to disperse the substance around each entry point uniformly.

Local anesthesia (2% lidocaine) was administered at the entry points for the knees, followed by the careful placement of a 25 g, 50 mm length cannula‐delivered product into the deep dermis. This infusion was directed toward the top of the knee joint.

Skin elasticity was assessed using the Pinch test, and photographic documentation was conducted before each treatment to evaluate wrinkle depth and skin laxity. A modified satisfaction questionnaire from the European Reference Network‐SKIN was administered to the patient before and after completing the treatment protocol to gauge satisfaction with the consultation, treatment prescription, therapeutic research, and overall satisfaction post‐treatment.

## 4 Case Report

**FIGURE 1:**
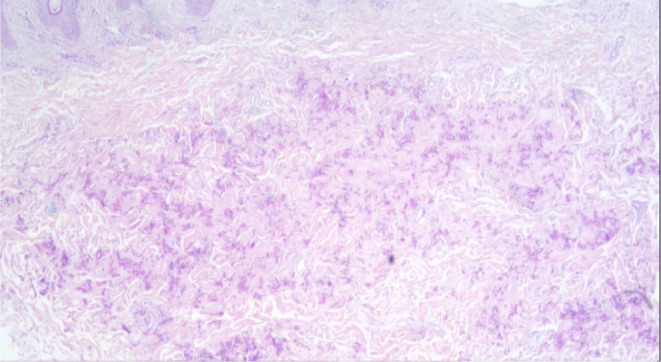
Skin biopsy from PXE's patient neck. Elastic fibers were irregular, thickened, fragmented, and haphazard in orientation in the mid‐dermis. Elastica VG and orcein stains verified their structure. Calcium deposition was identified by von Kossa stain.

A 27‐year‐old woman, diagnosed with PXE 5 years ago, visited the dermatology clinic in September 2023 to seek treatment for loose and sagging skin around her knees, neck, and armpits. The diagnosis of PXE was verified with a genetic test, fundus images, as well as a biopsy of her neck due to her progressive skin laxity in the cervical, axillary, and femoral regions (Figure 1). She had no pain complaints or inflammatory signs on her skin, and also she did not have any associated symptoms. She did not take any daily medication and had no relevant personal or family medical history of dermatosis. Finally, ophthalmoscopic investigation revealed the presence of angioid streaks alongside mottled pigmentation (peau d'orange).

Previously, she had tried various therapies, including fractional RF laser, equine collagen boosters, micro‐needling devices with platelet‐rich plasma and autologous exosomes, PDO threads, HA‐based skin boosters with peptides and amino acids, as well as PLLA. The results from all these treatments were temporary, lasting just for 1 month. The most significant effect was on the hydration of the skin, with less impact on skin tightening. In terms of side effects, the PLLA treatment caused itching and serious swelling on the thighs for 1 week, which was treated with anti‐histamine pills for 15 days. Also, small granulomas appeared in the inner thighs after 6 months. The fractional RF laser caused bruises that took about a month to fade away, and the equine collagen booster resulted in temporary reactive nodules for 1 week, which were treated with cortisone creams. Mild pain was experienced with PDO threads in the thighs, especially during movements, but eventually subsided. The patient experienced no side effects from the PRP, autologous exosomes, and skin boosters.

Although she experienced improved skin quality in her neck and armpits, she was dissatisfied with the results for her inner thighs and knees, which caused her significant stress.

Considering that the previous treatments the given patient has received, including other available bio‐stimulators, were all conducted by our team, we were highly interested in trying the new particle‐free PCL‐based bio‐stimulator to evaluate its potential as an alternative option for PXE improvement and comparing its results with the previous treatments. Observed advantages and disadvantages of each of the conducted treatments were thoroughly recorded and then summarized by the investigators' team (Table 1). The provided summary demonstrates that the particle‐free PCL injection is a possible alternative for PXE patients. No severe side effects or complications were observed during this study, and to the best of our knowledge, none had been reported worldwide, making this treatment a safe choice with sustainable results, considering the composition of the product, free of any micro‐particles.

**TABLE 1:** A comparative table of the treatments used to treat PXE in the selected patient.

Suggested treatments for pxe	Advantages	Disadvantages	Duration of tightening
Skin boosters, PRP	Immediate hydration of the skin, “plump” effect that gives the impression of skin tightening, no side effects or complications recorded	Short duration	15 days–1 month
Equine collagen	Improvement of skin texture	Short duration, inflammatory nodules lasted for 1 week	1 month
PLLA	Volumizing	Not impressive results in skin texture, serious swelling and itching for 1 week, granulomas formation after 6 months, more preparation and procedure time comparing to other treatments	3–4 months (only volumizing effect stayed for 6 months)
PDO threads	Immediate tightening effect, easy in use	Mild pain especially in movements, bruises	1 month
Ablative/non‐ablative lasers	Improvement of skin texture, immediate availability	Bruises for 1 month, erythema, pain, and itching for 15 days, skin peeling for 3 weeks	1 month
Liquid PCL	Gradual improvement of skin texture, tightening, and gives and a volume‐enhancing effect	Rare possibility of minor swelling after the treatment	At least 6 months (current follow‐up period)

The recommended protocol includes two sessions of particle‐free PCL, with eight syringes administered during each session and a two‐month gap between treatments. Photographic comparisons were taken before the first session, 2 months after the first session, and 2 months after the second session.

A detailed consultation was held to explain the post‐operative care protocol. The patient declined the prescription of methylprednisolone due to PXE disease, which mainly affects the gastrointestinal system. She was given oral antihistamine medication (cetirizine, 10 mg) 30 min before the treatment and instructed to continue taking it the following day. After the treatment, topical application of cortisone (ELOCON), arnica, and vitamin K creams, and a gentle massage were performed.

## 5 Results

Following the procedure, the patient displayed mild swelling without additional erythema and experienced pruritus on the same day. The deep injections in these relatively avascular anatomical structures utilizing cannulas resulted in substantial bruising, which disappeared within a week.

No long‐term adverse effects or complications have been registered 6 months following the treatment. The high safety profile of the fully liquid PCL, which contains no micro‐particles, can explain the difference compared to equine collagen and PLLA.

**FIGURE 2:**
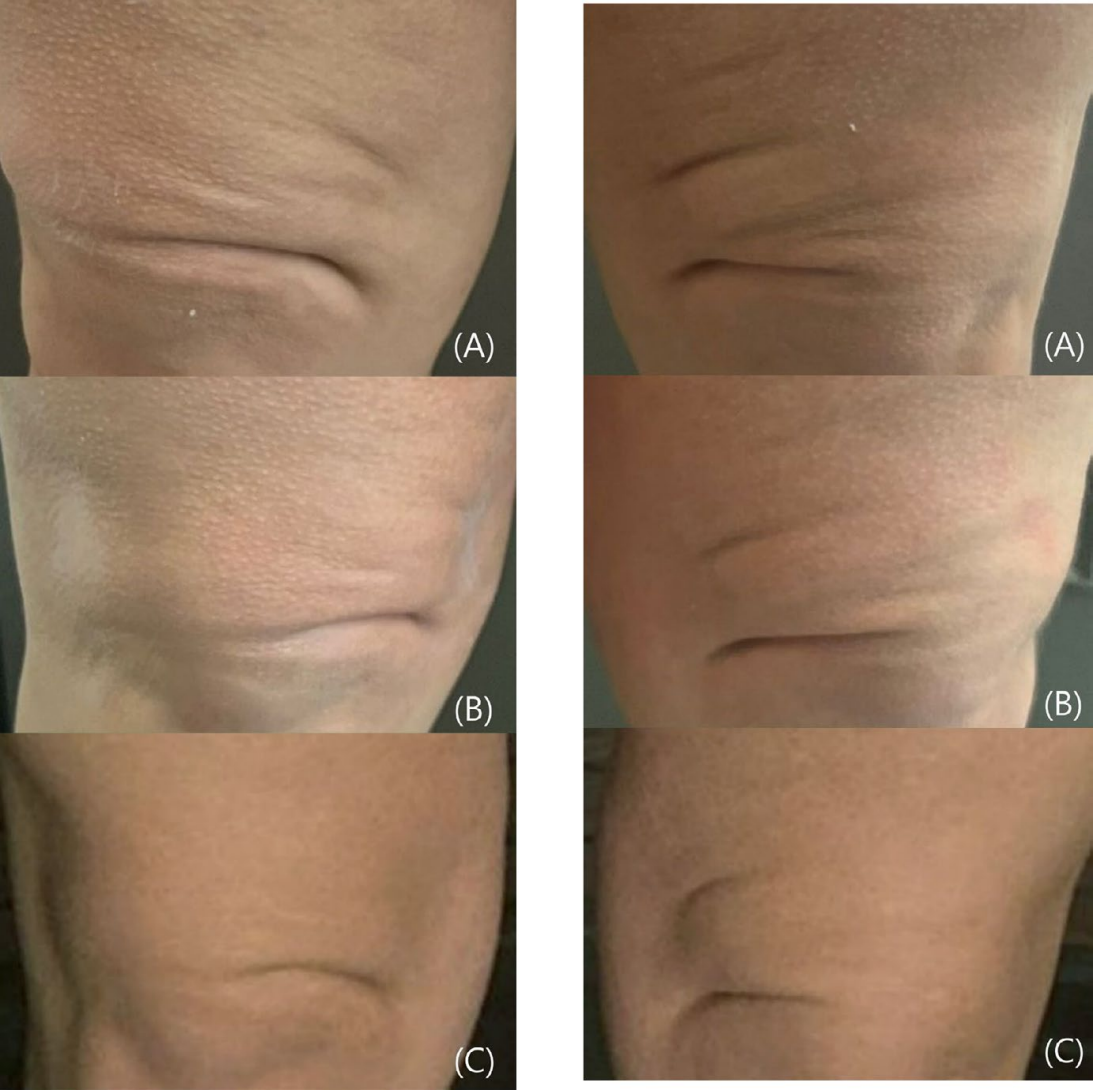
Knee (right and left) photographs of the PXE patient who underwent 2 injection sessions with particle‐free PCL. (A) Before the treatment, (B) 2 months after the first treatment, and (C) 2 months after the second treatment.

Two months after the initial treatment, photographic evidence displayed a noticeable reduction in the depth of the wrinkles surrounding the knees and an evident enhancement in skin tautness in the inner thigh area. Pinch test initially scored 4–5, and after the first session, it reduced to 3. 2 months later, the outcome following the second session was significantly more impressive, and the patient expressed high satisfaction levels with registered score 2 in Pinch Test (Figure 2). She reported perceiving an unprecedented amelioration in skin texture and experiencing a sense of firmness, distinct from her previous treatments. The patient expressed a positive willingness to undergo the treatment protocol after 6 months to sustain the achieved results. In the satisfaction questionnaire, the patient evaluated the entire protocol procedure (consultation, treatment, and follow‐up) with 5/5, expressing strikethrough her gratitude for the contribution of our team.

Six months after the patient has not yet repeated the protocol, and she remains satisfied with the result on her thighs and knees. The patient is under consideration to be enrolled in an FDA fast‐track clinical trial for ABCC6 deficiency. Long‐term follow‐up is planned in order to assess the durability of this treatment for at least one more year.

Fully liquid PCL does not have a direct volume‐enhancing effect like existing fillers. The injection process itself causes tissue repair. PCL, as a material, goes through three main phases: inflammation, proliferation, and remodeling. This includes the formation of granulation tissue and early appearance of collagen type‐III followed by long‐term collagen type‐I production and deposition during the remodeling phase. The implanted material in the cellular environment creates mechanical tension that stretches the fibroblasts, prompting them to produce collagen. In conclusion, the signals mentioned above create a suitable environment for the fibroblasts, highlighting the role of PCL as a foundation for new collagen and elastin formation [13]. Improving PXE's skin quality is based on restructuring the dermis. Therefore, the success of this treatment may be attributed to the combination of these signals that activate the fibroblasts in the deep dermis [14].

Fully liquid PCL appears to be more effective than other bio‐stimulators in improving skin quality in PXE, with longer‐lasting results. All published case reports worldwide have shown only temporary results and some registered side effects. This case report is limited to an individual patient, but the study's advantage is that this patient previously tried other treatments with less impressive and short‐lived results. Therefore, the reported results are promising for further studies, including larger clinical trials with a larger sample size of PXE patients and long‐term follow‐up.

Additionally, patients of different nationalities should be enrolled in multicenter studies conducted by large academic institutions and hospitals using a uniform protocol for the fully liquid PCL to facilitate the standardization of the PXE patient‐centered protocols. So far, more PXE patients have come in contact with our team, willing to try particle‐free PCL treatment for their skin manifestations. If a satisfactory number of patients are registered and the findings demonstrate broader applicability, this particle‐free PCL could become a promising official treatment for PXE globally.

## 6 Conclusion

The application of a liquid polycaprolactone collagen stimulator represents an efficacious method for promoting collagen synthesis in aged skin. Pseudoxanthoma elasticum (PXE) is a rare hereditary genetic disorder characterized primarily by dermatological manifestations, necessitating advanced cosmetic medical interventions to counteract cutaneous laxity and induce heightened production of collagen and elastin by hypoactive fibroblasts residing in the deep dermal layers. These preliminary outcomes from the aforementioned therapeutic regimen may benefit other PXE‐affected individuals who seek noninvasive modalities to enhance the quality of their skin. Further clinical studies should be encouraged to validate these findings and explore broader applications in dermatology. Finally, inspired by this case, this therapeutic regimen may be universally applied as a novel tool for rejuvenating skin in the aesthetic medicine field.

## Ethics Statement

The authors have nothing to report.

## Consent

The patient has provided written consent to publish her case, including using illustrative materials depicting her knees.

## Conflicts of Interest

The authors declare no conflicts of interest.

## Data Availability

The data that support the findings of this study are available on request from the corresponding author. The data are not publicly available due to privacy or ethical restrictions.
